# Recognition and treatment of severe sepsis in the emergency department: retrospective study in two French teaching hospitals

**DOI:** 10.1186/s12873-017-0133-6

**Published:** 2017-08-30

**Authors:** Philippe Le Conte, Séverin Thibergien, Jean Batiste Obellianne, Emmanuel Montassier, Gilles Potel, Pierre Marie Roy, Eric Batard

**Affiliations:** 1 Service des urgences, CHU de Nantes, 44035 Nantes cedex 01, France; 2 Service des urgences, CHU d’Angers, 49000 Angers cedex, France

**Keywords:** Severe sepsis, Emergency medicine, Surviving sepsis campaign, Bundles, Fluid loading, Recognition

## Abstract

**Background:**

Sepsis management in the Emergency Department remains a daily challenge. The Surviving Sepsis Campaign (SSC) has released three-hour bundle. The implementation of these bundles in European Emergency Departments remains poorly described.

The main objective was to assess the compliance with the Severe Sepsis Campaign 3-h bundle (blood culture, lactate dosage, first dose of antibiotics and 30 ml/kg fluid challenge). Secondary objectives were the analysis of the delay of severe sepsis recognition and description of the population.

**Methods:**

In accordance with STROBE statement, we performed a retrospective study in two French University Hospital Emergency Departments from February to August 2015. Patients admitted during the study period were screened using the electronic files of the hospital databases. Patient’s files were reviewed and included in the study if they met severe sepsis criteria. Demographics, comorbities, treatments were recorded. Delays from admission to severe sepsis diagnosis, fluid loading onset and antibiotics administration were calculated.

**Results:**

One hundred thirty patients were included (76 men, mean age 71 ± 14 years). Blood culture, lactate dosage, antibiotics and 30 ml/kg fluid loading were performed within 3 hours in % [95% confidence interval] 100% [96–100%], 62% [54–70%], 49% [41–58%] and 19% [13–27%], respectively. 25 patients out of 130 (19% [13–27%]) fulfilled each criteria of the 3-h bundle. The mean fluid loading volume was 18 ± 11 ml/kg. Mean delay between presentation and severe sepsis diagnosis was 200 ± 263 min, from diagnosis to fluid challenge and first antibiotic dose, 10 ± 27 min and 20 ± 55 min, respectively.

**Conclusion:**

Compliance with SSC 3-h bundle and delay between admission and sepsis recognition have to be improved. If confirmed by other studies, an improvement program might be deployed.

## Background

Severe sepsis and septic shock represent the most severe forms of infection. Two consecutive epidemiological surveys demonstrated an increasing incidence for severe sepsis [1, 2] from 2000 to 2012; it accounted for 500,000 annual emergency visits in USA in 2006 [3]. During the same period, attributable mortality rate declined from 40% in 2000 to 18% in 2012 [2].

Severe sepsis was defined in 2001 by a consensus conference as a presumed infection with a systemic inflammatory response syndrome (SIRS) and almost one organ dysfunction [4]. Although this definition had some weaknesses, it was used during almost two decades. More recently, a new consensus conference (SEPSIS-3) modified sepsis definition with introduction of screening by the quick SOFA (qSOFA) [5].

In their famous paper, Rivers et al. demonstrated that an intensive treatment during the first 6 hours after triage decreased mortality [6]. Since 2004, several recommendations from the Surviving Sepsis campaign (SSC) have been released [7] with objectives at three and 6 hours after triage. Objectives at 3 hours are: obtain lactate level and blood culture, administer broad spectrum antibiotics and administer 30 ml/kg crystalloid in case of hypotension or lactate ≥4 mmol/l. The implementation of theses guidelines has proved to decrease mortality from 37 to 30% [8].

However, the implementation of the SSC bundles in overcrowded Emergency Departments (ED) - in particular volume and delays of fluid loading and first antibiotic administration - remains difficult [9]. The early recognition of severe sepsis by the triage nurse is a real challenge because the clinical presentation might be confusing. This low compliance was demonstrated in Australia [10].

These facts motivated us to conduct a retrospective study of the compliance with the SSC 3 h-bundle and of the delay of recognition of severe sepsis in two EDs of French teaching hospitals. This study was realized before SEPSIS-3 release, thus the previous severe sepsis definition was used. Primary output was compliance with 3 h-bundle, secondary were analysis of the delay of severe sepsis recognition and description of the population.

## Methods

This study was designed in accordance with STROBE statement [11].

### Setting

This retrospective study was performed from February to August 2015 in the EDs of two teaching Hospitals located in west of France. Both were staffed 24 h a day by senior Emergency Physicians, residents and one or two triage nurses depending on the patients flow. The two ED were equipped by an electronic system including all time stamp clinical data. The triage derived from the Canadian scale were comparable with the same number of categories. Their annual volume were 75,000 and 50,000 adult patients. They belonged to University Hospitals with all specialties including intensive care units. Clinical management was not altered during the study period and there was no written local guidelines recommending delays for management of sepsis in the ED.

## Patients and methods

### Patients

Patients older than 18 years and presenting a severe sepsis as defined by the 2001 consensus conference [4] were included. Theses criteria were: suspected or documented infection and at least two of the four SIRS criteria (temperature < 36 °C or >38 °C, heart rate > 90 B/min, tachypnea >20/min, white blood count <4000/mm3 or >12,000/mm3) and at least one organ failure. Patients for whom another diagnosis than sepsis (pancreatitis, cardiogenic pulmonary edema…) was made at the end of the hospitalization were excluded.

### Methods

Patients were screened using the databases of the two hospitals from February to August 2015. Patient’s files were reviewed and included in the study if they met severe sepsis criteria. For each included patient, relevant data were exported and stored in a Microsoft Access Database (Seattle, WA).

The following data were recorded:

- Demographics, major comorbities (cancer, cardiac and respiratory insufficiency, immunosupression, diabetes), autonomy evaluated by the Knaus classification [12], underlying disease evaluated by Mac Cabe classification [13].
- Hospital management including triage priority by the nurse, delays between admission, sepsis diagnosis, administration of the first dose of antibiotics, administration and volume of fluid challenge were recorded. All the delays were calculated using extracted patient’s time stamp electronic files.
- Outcomes defined as admission in intensive care unit (ICU), length of stay in the hospital and mortality were recorded.
- Compliance with 3-h SSC bundle was calculated for each patient taking into account blood culture and lactate samples done, delay of antibiotic administration <3 h, delay of fluid loading <3 h and volume > = 30 ml/kg.

### Ethics

As it was a retrospective study, according to the French legislation (articles L.1121–1 paragraph 1 and R1121–2, Public health code), the approval of the ethics committee was not needed to use anonymous data for retrospective observational study. Furthermore, same articles stated that ethic approval was not needed to access to hospital databases by medical investigators for a research purpose

### Statistical analysis

Anonymized data were analyzed using PASW statistics (SPSS, USA). Patient characteristics were described using mean and standard deviation (SD) for continuous outcomes, and numbers and percentages with 95% confidence interval for qualitative ones. Continuous data were compared using Student’s t test or Mann Whitney if necessary and proportions were compared by Khi2. A *p* value <0.05 was considered significant.

### Number of patients

For an overall compliance to 3-h SSC bundle of 30% compared to 45% in the literature, in bilateral formulation, with alpha risk 0.05 and beta 0.10, the number of patients was 109.

## Results

### Patients characteristics

One hundred thirty patients were included, 76 men and 54 women, mean age 71 ± 14 years. Baseline characteristics are displayed in Table 1.

**Table 1:** Patient’s characteristics and severe sepsis criteria in 130 patients admitted to an ED with severe sepsis

Variables	N (percentage) [95%CI]
Place of living	
Home	114 (88%) [80–92%]
Long term care facilities	16 (12%) [7–19%]
Number of comorbities	
0	40 (31%) [23–39%]
1	38 (30%) [22–37%]
2	33 (25%) [18–33%]
3	15 (11%) [7–18%]
> 3	4 (3%) [1–7%]
Major Comorbities	
History of cancer	33 (25%) [20–32%]
Heart failure	31 (24%) [8–32%]
Immunosupression	27 (21%) [14–28%]
Diabetes	26 (20%) [14–28%]
Chronic pulmonary disease	14 (10.7%) [6–17%]
Mac Cabe classification	
0	37 (28%) [21–36%]
1	47 (36%) [28–44%]
2	27 (21%) [14–28%]
ND	19 (15%) [9–22%]
Knaus classification	
1	67 (51.5%) [43–59%]
2	33 (25.5%) [19–33%]
3	24 (18%) [13–26%]
4	6 (5%) [1–10%]
SIRS variables	
Fever or hypothermia	100 (77%) [69–83%]
Tachycardia	96 (74%) [66–81%]
Tachypnea	70 (53%) [45–62%]
Hyperglycemia (except diabetes)	24 (18%) [13–26%]
Altered mental status	27 (21%) [15–29%]
Hyperleucocytosis	89 (68%) [60–76%]
Organ failure (for severe sepsis diagnosis)	
Hypotension	108 (83%) [76–89%]
Hypoxaemia	47 (36%) [28–45%]
Rise in creatinine	73 (56%) [46–64%]
Thrombopenia	31 (24%) [17–32%]
Lactates	77 (59%) [51–67%]
Priority after triage	
1	2 (1.5%) [0.3–4%]
2	18 (14%) [9–21%]
3	66 (51%) [42–59%]
4	42 (32%) [25–40%]
ND	2 (1.5%) [0,3–4%]

### Compliance with three-hour SSC bundle

Results for the four items are displayed in Table 2.

**Table 2:** compliance with SSC three hours bundle

SSC three hours bundle	Patients (%)
Blood culture	130 (100% [96–100%])
Lactate dosage	81 (49% [54–70%])
Antibiotic delay <3 h after triage	64 (49% [41–58%])
30 ml/kg fluid loading <3 h after triage	25 (19% [13–27%])

Overall, 25 patients out of 130 (19% [13–27%]) fulfilled the four criteria. The principal missing criteria was the fluid loading volume (18 ± 11 ml/kg) while the SSC 3 h-bundle was 30 ml/kg. Prescribed antibiotics were broad-spectrum in 111 patients (85% [78–91%]).

### Delays

Mean time between ED admission and severe sepsis diagnosis was 200 ± 263 min, between diagnosis and fluid challenge 10 ± 27 min and first antibiotic injection 20 ± 55 min, respectively. Overall, the actual time from triage to first medical contact was consistent with that provided by the triage scale in 42 patients (32% [25–42%]). Delay from ED admission and ward admission was 738 ± 605 min. The hospital length of stay was 12.8 ± 18.1 days.

### Outcomes

Seventy four patients (57%) were admitted to an Intensive Care Unit (ICU), 49 (38%) to a medical or surgical ward and 7 (5%) died in the ED. Infectious foci are displayed in Table 3.

**Table 3:** Final infectious foci in 130 patients admitted to ED with severe sepsis

Infectious foci	Number (percentage)
Urinary	40 (30.7%) [23–39%]
Pulmonary	37 (28.5%) [21–36%]
Abdominal	18 (13.8%) [9–20%]
Others	15 (11.5%) [7–18%]
undetermined	9 (6,9%) [3–13%]
cutaneous	5 (3,8%) [1–8%]
Meningitis	3 (2.4%) [0.4–6%]
Febrile neutropenia	3 (2.4%) [0.4–6%]

Noradrenaline was prescribed in 21 patients in a delay of 2.5 ± 2.1 h after sepsis diagnosis. In-hospital mortality was 29% [95%IC 22–37%]. The mortality was not different between patients admitted to a ward when compared to those admitted in ICU (24% versus 25% respectively, p 0.9). There was no significant differences in patients characteristics nor delays between the two EDs.

## Discussion

Few real-life studies have explored the compliance to the SSC bundles in European EDs. Our main result was the low proportion (19%) of patients who were treated in accordance with the 3-h sepsis bundle [7]. This proportion was similar to one previously reported in Scottish EDs (between 29 and 43%) [14]. Outside Europe, compliance to SSC bundles was comparable to our study, 29% in Australia [10] and 28% in USA [15].

The low compliance with the 3-h SSC bundle was mainly due to low volume of fluid loading (18 ± 11 ml/kg) when compared to the recommendations (30 ml/kg) [7]. This volume was comparable to that of the control group in an ED trial [16]. The advanced age and the presence of cardiac comorbities might explain this result because physicians might fear fluid overloading. Our patients were older than those included in the ARISE trial (71 vs 63 years) and the incidence of cardiac comorbity was higher (24 vs 10%) [17]. Similar results were reported in a European ICU severe sepsis population [18]. A non invasive approach using bedside cardiac and lung ultrasound has demonstrated its usefulness and is recommended by a consensus conference [19]. Firstly, a Point-of-Care Ultrasound could affirm the diagnosis of severe sepsis when faced to an undifferentiated shock [20]. Secondly, index of hypovolemia like left ventricular obliteration [21] and/or collapsed inferior vena cava [22] could secure initial fluid loading. Thirdly, after fist infusion, dynamic indices of fluid responsiveness such as passive leg rising [23] could be used along with lung ultrasound for tolerance [24].

The poor compliance to the 3-h SSC bundle is partly explained by the delay between admission and severe sepsis recognition (more than 3 h). Although priorities after triage indicated a need for first medical contact within 120 min and 180 min, this delay was respected in only 32%. Although not constant, overcrowding of these two EDs was frequent. This fact has been associated with a poor compliance with SSC bundles [9]. A systematic review has explored the efficacy of electronic systems for the diagnosis of severe sepsis and did not find any improvement of survival [25]. A real-time electronic surveillance in the ED decreased time to blood culture but not to antibiotic administration [26]. A complex quality improvement project increased the adherence to sepsis bundles from 28% to 71% in a US ED [15]. A multicenter improvement program in 97 Australian EDs increased overall survival through the implementation of a screening tool and sepsis bundles [10]. All these studies included an enhanced triage process and simple guidelines (e.g. antibiotic administration in less than 1 hour after sepsis recognition). The implication of all health-care workers (nurses, physicians) was mandatory along with the implementation of electronic alerts [10, 16]. Finally, the quick SOFA, a simple bedside clinical score recommended by the SEPSIS-3 consensus conference, may improve the screening of sepsis in the ED [5].

### Limitations

This study has three limitations. First, it was conducted before SEPSIS-3 publication, thus old definition of severe sepsis was used. Second, due to its retrospective nature, all the clinical features could not be retrieved. However, the two EDs have electronic time stamp files allowing recovery of the majority of them. Thirdly, this study included only two teaching hospital EDs and it is possible that the described population does not reflect the situation of all French EDs.

## Conclusion

There is a poor compliance with the 3-h SSC bundle in the ED and delay between admission and severe sepsis diagnosis has to be improved. This French situation is comparable to that observed elsewhere before implementation of new sepsis protocols. If confirmed by other studies, this situation should motivate the emergency physicians to improve their practice. A complete process involving nurses and physicians education, electronic sepsis alert, enhanced triage process and diffusion of simple sepsis bundle could be deployed. Societies of Emergency Medicine could play an incentive role in this process.

### What this paper adds

#### What is already known on this subject

Severe sepsis, the most severe form of infection, has a growing incidence and account for 500,000 annual Emergency Department (ED) visits in USA. Compliance to the Surviving Sepsis Campaign (SSC) bundles and shortening delay of recognition in busy EDs remain challenging. Little is known in European EDs.

#### What this study adds

Both compliance to SSC bundles and delay of recognition in representative French EDs were not satisfying. In particular, fluid loading volume was far under this recommended by the SSC. Delay from triage to sepsis recognition was over 3 h. A complete improvement process would be deployed in order to enhance patient’s survival.
